# Integrated understanding of hydrocephalus — a practical approach for a complex disease

**DOI:** 10.1007/s00381-021-05243-3

**Published:** 2021-06-10

**Authors:** U. W. Thomale

**Affiliations:** Pediatric Neurosurgery, Charité Universitätsmedizin, Berlin, Germany

**Keywords:** Hydrocephalus, Infants, Pathophysiology, Classification, Shunt, Neuroendoscopy

## Abstract

Most of childhood hydrocephalus are originating during infancy. It is considered to be a complex disease since it is developed on the basis of heterogeneous pathophysiological mechanisms and different pathological conditions as well as during different age groups. Hence, it is of relevant importance to have a practical concept in mind, how to categorize hydrocephalus to surgically better approach this disease. The current review should offer further basis of discussion on a disease still most frequently seen in Pediatric Neurosurgery. Current literature on pathophysiology and classification of pediatric hydrocephalus has been reviewed to integrate the different published concepts of hydrocephalus for pediatric neurosurgeons. The current understanding of infant and childhood hydrocephalus pathophysiology is summarized. A simplified concept based on seven factors of CSF dynamics is elaborated and discussed in the context of recent discussions. The seven factors such as pulsatility, CSF production, major CSF pathways, minor CSF pathways, CSF absorption, venous outflow, and respiration may have different relevance and may also overlap for the individual hydrocephalic condition. The surgical options available for pediatric neurosurgeons to approach hydrocephalus must be adapted to the individual condition. The heterogeneity of hydrocephalus causes mostly developing during infancy warrant a simplified overview and understanding for an everyday approach. The proposed guide may be a basis for further discussion and may serve for a more or less simple categorization to better approach hydrocephalus as a pathophysiological complex disease.

## Introduction

Hydrocephalus is a condition of CSF circulation disturbance with imbalanced CSF flow dynamics of various reasons and develops as a symptom of an underlying disease. According to our database of more than 500 primary surgeries, more than 60% of pediatric hydrocephalus need their first surgical treatment during infancy, while approximately 74% of shunted patients have received surgery during infancy at an age younger than 24 months [unpublished data]. The causes of hydrocephalus are heterogeneous such as the pathophysiologic mechanism leading to disturbed CSF flow. Many authors have previously described how to classify hydrocephalus which led to different concepts [18, 27, 36, 55, 57, 61, 65]. The first well-known concept was developed by Walter Dandy which based on observations to apply a dye into the lateral ventricle and perform a lumbar puncture in patients with proven hydrocephalus. In case he identified the dye also in lumbar puncture, he classified the condition as communicating hydrocephalus, while in cases when the dye was not seen in lumbar CSF, a non-communicating hydrocephalus was concluded [18]. Despite a relevant progress which was made during the last century, Dandy’s classification remains still important because his concept remains our basis for decision making on how to treat the hydrocephalus either with a shunt in communicating conditions or by endoscopic fenestration to reestablish communication of CSF between internal and external CSF spaces in non-communicating hydrocephalus. In contrast to that, Shizuo Oi established a multi-categorical classification that has taken different categories and subtypes of hydrocephalus into account, mainly patient characteristics, CSF pathophysiological conditions, and treatment options. This classification defined hydrocephalus as a complex disease given a theoretical number of more than 75 million subtypes of hydrocephalus [55]. This scheme is of high importance to take all factors involving the development of hydrocephalus into consideration and to give a better understanding of different hydrocephalus conditions, but it may be less applicable in daily clinical routine. Other milestones of hydrocephalus understanding include the “bulk flow” concept by Russel, the “pulse pressure” induced hydrocephalus by Di Rocco and the hydraulic circuit by Rekate [19, 65, 69]. In recent years, further understanding of different driving forces for CSF flow is discussed. Those include to distinguish between minor and major CSF pathways [78]. Also, pathophysiology hypotheses according pulsatility and respiration were described [2, 27, 61], which have questioned historical understanding of CSF flow. These critics have also been observed in experimental work of lacking aqueductal CSF outflow by Oreskovic and coworkers [56]. Nevertheless, research on molecular level of cellular and microvascular CSF transport is an utmost important basis for the understanding of CSF dynamics [1, 33, 54]. All these approaches for a better understanding of physiology and pathophysiology are intensely discussed in recent literature [36]. It may be useful to further develop on this and to integrate the way of understanding from different theories to a more holistic approach toward hydrocephalus. The main question behind all this may be, if the development of CSF circulation disturbances can be explained by only one theory or if different theories are valid but account in variable intensities in different individual circumstances of hydrocephalus. The present report tries to integrate different ideas of hydrocephalus pathophysiology and develop a model for a practical understanding of a complex pathophysiological condition.

### Anatomical consideration

Rekate and colleagues described the hydraulic circuit of CSF as a continuum of the arterial inflow; the CSF production; the CSF flow along the ventricles, cisterns, spinal, and cranial subarachnoid space; and finally the venous outflow [65]. One relevant driving force for this circuit is the heart rate. Within this CSF circuit, Oi’s definition of major CSF pathways in contrast to the minor CSF pathways may be integrated [78]. The major CSF pathways are well identifiable by MRI. It is observed that the blockage of the foramina of Monro might lead to an isolated lateral ventricle if located on one side or a biventricular hydrocephalus if both sides are affected. The aqueductal stenosis, which might actually be a misleading terminology because, in more cases, we are observing a complete occlusion rather than a stenosis, may lead to a triventricular hydrocephalus, while the occlusion of the outlets of the 4th ventricle may result in ventriculomegaly of all four ventricles. All those conditions are classically linked with a pressure gradient between internal and external CSF spaces with narrowed subarachnoid space on the convexity or if the aqueduct or any distal structures are affected a bulging of the membranes at the 3rd ventricle (lamina terminalis, floor of the third ventricle or pineal recess) [20, 37]. Those forms of hydrocephalus will still account for the classic nomenclature of non-communicating hydrocephalus as introduced by Dandy and are having the implication that endoscopic treatment will be well indicated in order to reestablish communication of CSF flow [18]. Success of endoscopic ventriculo-cisternostomy (ETV), however, further depends mainly on age and the underlying disease leaing to hydrocephalus [43, 47, 67]. Recently, another form of non-communicating hydrocephalus was introduced, which is called either panventriculomegaly, external occlusive hydrocephalus, or prepontine occlusive hydrocephalus [3, 25, 35] and is defined mainly by a prepontine obstruction of CSF spaces in which the CSF communication toward the subarachnoid space of the convexity is blocked. If this condition is observed during infancy (age < 1 year), similarly, ETV could not successfully be applied in contrast to older children [3]. This clinical picture may be similar to the experimental Kaolin model, in which the injection into the basal cisterns leads to hydrocephalus [26].

Additional anatomic relevant structures are the correlates of CSF production and CSF absorption. Those have also been discussed extensively, and it turned out that both mechanisms are based on a variety of anatomical structures [29, 33, 41, 52, 57, 62]. The CSF production is thus been postulated to take place not only in the choroid plexus but also in any arterioles and capillaries within and on the surface of the cerebral tissue [59]. The absorption of CSF seems to be even more complex and is described to take place not only in arachnoid villi, also known as Pacchionian granulation, but also in the venules and capillaries at the surface and inside the cerebral tissue as well as toward the lymphatic system within the dura, any nerve roots pockets, or the cribriform plate [58].

Also, the foramen magnum seems to be relevant for balancing any pulsation from the cranial space toward the spinal canal since the calvarium represents a firm cavity without any ability to balance out pulsatile activity externally while the spinal canal’s dura is surrounded by epidural fat, which enables elasticity and a thus offers a “Windkessel” function to somehow flatten the pulsatile curve [51].

### Pulsatility

Theories of hydrocephalus development due to increased pulsatility and decreased compliance have first been indicated by Di Rocco, who inserted a cardiac rhythm synchronized pulsating balloon in the ventricle, which resulted in ventricular enlargement without direct occlusion of any major CSF pathways [19]. The interpretation was found in direct impact of pulsatile peaks of ICP to the CSF spaces without increasing mean ICP values resulting in induced disruption of intracranial compliance. Later, the hydrodynamic theory described the close interaction of rhythmic heart-driven arterial blood inflow into the intracranial space in more detail [28, 29]. The elasticity in the arterial wall leads to direct pulsation amplitude toward the brain parenchyma and the internal and external CSF spaces. Due to the temporal profile of the pulsation in the different compartments, a direct occluding effect on the subarachnoid bridging veins is postulated, hindering the venous outflow toward the sinuses. In parallel, the transmitted pulse wave to the CSF space will be transiently increased, enabling a better pressure gradient for adsorption of CSF back to the venous as well as lymphatic system [28, 29]. If this timely balanced pulsatile system is disturbed by various factors such as altered elasticity of the arterial wall, cerebral tissue composition, or occlusion of the foramen magnum, the pulsatile amplitude might increase and may result in harmful effects of pulse wave–induced ICP peaks toward the brain parenchyma with possible secondary chronic enlargement of the ventricular system. The theory of Greitz was further evolved by Preuss and colleagues [61]. It was postulated that the pulse wave within the intracranial arterial system will lead to a centripedal pressure force from the brain tissue convexity which is directed toward the center and induces an interstitial fluid shockwave to the ventricular system. The pressure wave which is induced in the ventricles may be stronger than the CSF volume which can be drained through the foramen of Monro and the Sylvian aqueduct resulting in a reflection of a centrifugal pulse wave as well as an interstitial flow of CSF toward the subarachnoid spaces. In addition, the increase in blood volume induces volume changes in the subarachnoid space with a frontal occipital direction. Both factors induce CSF pulse waves within the subarachnoid space which is finally reached the convexity during the diastolic arterial pulse wave. Additional venous drainage and the resulting decrease in venous pressure enable transcapillary CSF absorption. This finely balanced interplay of different pulse directions leads to a pulsatile but ongoing interstitial as well as ventricular and extraventricular fluid movement translated somehow into CSF flow. The concept is summarized as the pulsatile vector theory, in which the arterial cerebral perfusion and the venous outflow dynamics are a relevant driving force of intracranial CSF circulation. Any disturbance of this balance may lead or contribute to pathophysiological CSF dynamics of different hydrocephalus conditions [61]. These theories however may underestimate the role of the intraspinal CSF space and its role for CSF circulation.

### Spinal CSF flow and body posture

Recent advances in MR imaging have enabled to investigate CSF flow within different anatomical levels of the CSF space. Initially, CSF pulsation sequences directed a lot of intention toward pulsatile flow characteristics within the Sylvian aqueduct [11]. Further investigations with more conclusive information were reached by time-SLIP sequences. These investigations were visualizing pulsating flow velocity signals at the foramen of Monro, the aqueduct, and the prepontine cisterns and only little or even no flow signals in the ventricular bodies and the cranial convexity [82]. New insights were gained in the spinal CSF spaces, which resulted in altering CSF flow characteristics in prone or supine positions. Pulsatile flow was observed in the upper part of the spinal CSF space but not in the lower part in each respective body position, which was interpreted by the location of less resistance to flow in the upward compartment [82]. In addition, a pulsatile flow was observed throughout the spinal CSF spaces with pulsatile characteristics. Further insights were described by real-time phase-contrast flow MR imaging investigating respiration-related spinal CSF flow patterns [2, 21]. The studies were performed in supine position using a respiration protocol of 4 cycles with forced in- and expiration phases. An inspiration-associated cranial CSF flow was described which is more pronounced in the cervical levels of the spine compared to the thoracic and lumbar levels. The downward movement of CSF was seen in association with expiration patterns but mostly pronounced in the lower spine. A watershed area was postulated to be located at the level of the heart with dominated upward CSF net flow in the levels from C3 to TH1, while a downward CSF net flow was observed at the levels between TH8 and TH12. The driving force of CSF flow in the spinal canal during respiration is triggered by changes in epidural venous filling which might be called the venous respiratory pump [21]. These patterns were confirmed in another study protocol comparing thoracic to abdominal breathing patterns. In general, the abdominal breathing showed higher flow volumes during inspiration and expiration phases. At all spinal levels, an upward CSF net flow was calculated which was non-significantly higher in abdominal breathing pattern. At the level of the aqueduct, almost no net flow was observed in the expiration phase with minimal upward net flow in the inspiration phase [2]. These surprising observations were further evaluated in surgical conditions looking at flow patterns during endoscopic intervention videos, which showed in 85% of the cases an inspiration synchronous upward CSF flow at the stoma [12]. These recent studies underline the importance of respiration for spinal CSF flow which might have been underestimated so far. However, most of the investigations have been performed in supine horizontal position of the patients. As mentioned above, Yamada and coworkers observed positional-dependent differences in CSF flow patterns for supine and prone positions in the spinal CSF spaces. These were also observed by Lee and coworkers in rodent CSF dynamic contrast studies, who observed that lateral horizontal position showed enhanced clearance capabilities of the glymphatic transport [48]. Very limited studies have been performed in upright position. Alperin and coworkers have investigated blood flow as well as CSF flow in horizontal and upright body positions using cine phase-contrast MR imaging. Significant different patterns of venous outflow and oscillatory CSF flow in the upright compared to supine position combined with a decrease of ICP and an increase of compliance in the upright position are described [4, 5]. They hypothesized that the shift of mainly venous blood out of the cranium as well as the CSF, which is partially shifted in the spinal canal, occurs during the transition from the supine toward the upright position. To reduce intracranial volume outflow, the jugular veins are known to collapse in upright position. The displacement of CSF is explained by the expandable spinal dural cavity and will increase in volume in upright position due to hydrostatic pressure [5]. The CSF volume shift in different body postures was already proven by Bjoern Magnaes as published in 1989, who implanted a lumbar needle and a ventricular catheter connected by tubing system as external craniospinal shunt in a few patients [51]. He was able to measure posture-dependent pressure as well as flow changes in lying and upright position. His observations revealed predominantly cranial pulsatile CSF movement in lying position and caudal pulsatile CSF movement in upright position [51].

### Minor CSF pathways

Oreskovic has questioned any net CSF flow at the aqueduct level after performing the cannulation of the aqueduct and connecting the tube to an external drain and failing to observe any drainage of CSF through the cannula at the same level of the aqueduct [40]. This fact was interpreted that unidirectional flow of CSF is not present in the CSF spaces but pulsatile flow takes place in basically all directions, and interstitial CSF flow does additionally play a relevant role in CSF exchange. Virchow Robin Spaces, intraparenchymal capillaries, and the ependymal and the choroid plexus are relevant minor pathways for CSF to enable ventricular as well as extraventricular transparenchymal CSF exchange. In this context, it was postulated and also experimentally observed that CSF secretion does not only take place within the choroid plexus. In addition, an ultrafiltration of fluid in the parenchymal arterioles and capillaries with high capillary pressure is another source of CSF production [41, 59]. Similarly, the absorption of CSF takes place not only in the arachnoid villi but also in the intraparenchymal venules and capillaries with low hydrostatic pressure as well as toward the transition zones to the lymphatics at the lamina cribrosa and nerve root dural interfaces [31, 64]. Since the capillary system in the brain parenchyma is postulated to be 5000-fold larger than in the choroid plexus, the parenchyma plays a significant role for keeping the water exchange balance. The blood–brain barrier represents a unique blood tissue interface in the human body. Transcapillary water transport has been reported to be established by mainly aquaporin 1 channels in the ependymal and choroid plexus but also aquaporin 4 channels in the astrocytic basal membranes. In addition, unidirectional water and ion exchanges does also rely on osmotic gradient [1]. On the ependymal surface, ciliary activity has benn identified, which may play an additional role for microtransport of CSF along the ependymal surface but will need further investigation in the future [68, 80]. The water content within the brain parenchyma is at about 80–90%. Beside intracellular and intravascular water content, the main portion of water is located in the interstitial space, and the transport in the parenchyma may mainly take place in the perivascular Virchow Robin spaces to enable the transport of nutrition and wash out any waste such as β-amyloid. This transport may be from arterial capillary to parenchyma to venous capillary. It might also involve CSF transport into or out of the ventricles or the external CSF spaces. The driving forces of CSF transport in the capillary and interstitial compartments are described to be the hydrostatic and the osmotic pressure [1, 32, 34, 54, 58, 59].

### Venous congestion

The absorption of CSF takes place through different anatomical correlates of venous outflow and lymphatic system. Any venous congestion due to different reasons of pulmonary hypertension, right heart failure, venous outflow stenosis, or cranial constriction, has direct consequences on CSF dynamics. The venous absorption is impaired, but in parallel, the intracerebral blood volume is increased. Classically, the idiopathic intracranial hypertension, formerly known as pseudotumor cerebri, is associated with increased intracranial pressure with narrow ventricles [8, 49, 53, 76]. However, CSF release will improve the patient's condition by interrupting the vicious cycle that venous congestion is partially released also by decreasing the external CSF pressure and thereby the compression of bridging veins. In infancy, this clinical condition is known as macrocephaly, subarachnomegaly, or benign hydrocephalus [70]. Mostly, the transition of increased CSF absorption by development of arachnoid villi will always cause some impaired absorption period during infancy. Some patients will show an prolonged macrocephaly, which might resolve during further growth of the cranial base and thereby improving venous outflow through jugular veins and the cavernous sinus. Only very few of those patients will need further attention to decide if a CSF diversion procedure is needed [72].

### Particularities in infant hydrocephalus

Developmental aspects are well known to contribute to understanding of CSF physiology. The choroid plexus develops in the embryo, but CSF did already exist being produced in capillary fluid exchange of other origin [63]. Moreover, arachnoid granulations are not present in early infancy, and a transition of CSF absorption will develop in the first months of life. Maybe the most striking anatomical difference during infancy is the soft calvarial cavity with an open fontanelle enabling elastic volume changes of the cranial compartment which will disappear mostly in the first year of life. It has direct implication for volume exchange between blood volume and CSF volume as well as exchange of CSF volume between the intracranial space and the spinal column. Harold Rekate has recently put special emphasis on hydrocephalus pathophysiology in infancy [66]. Two of three factors are especially relevant to consider in the treatment of infant hydrocephalus: firstly, rapid brain growth with the development of the mature nervous system in the context of distortion by the ventricular distension, and secondly, the distensible nature of the skull without a fixed intracranial volume [66]. Two obvious examples emphasize these differences. One is the fact that ETV does not show the same effectiveness in treating non-communicating hydrocephalus due to aqueductal stenosis or 4th ventricular outlet obstruction during infancy compared to older children [47]. The other is that there are different consequences observed for venous congestion leading to macrocephaly and subarachnomegaly together with ventricular enlargement during infancy when the sutures are active and the fontanelle is open in contrast to idiopathic intracranial hypertension which develops in circumstances of a closed calvarium, and the CSF spaces remain narrow most likely due to increased intraparenchymal blood volume [8, 70]. It was further emphasized that a certain amount of ICP facilitates CSF flow toward the cortical subarachnoid space and enables the shift of CSF into the venules and arachnoid villi if already present. On the basis of this theory, Epstein and Hochwald postulated that a moderate compression band applied to posthemorrhagic hydrocephalic infants could increase the absorption capacity of CSF and lead to normalization of CSF circulation disturbances [22]. On the other hand, it might also lead to unwanted ICP increase and pressure-induced developmental disturbances if the absorption capacity will not be influenced positively. The dynamic head growth during infancy due to hydrocephalus crossing the percentiles of normal growth upwards can, however, be reversed by putting a shunt system. This may be a very good parameter to adapt physiologic-like CSF drainage during shunt therapy for adjustment protocols adapting the drainage resistance in the valve system. The aim would then be to keep the percentile curves in order to avoid over- as well as under-drainage and keep a physiological pressure value which seems more relevant than a certain ventricle size [24, 30]. Such a strategy if successfully applied would automatically avoid slit ventricle syndrome and its complications in the long run.

### Seven pathophysiological categories and considerations on hydrocephalic entities

**Fig. 1:**
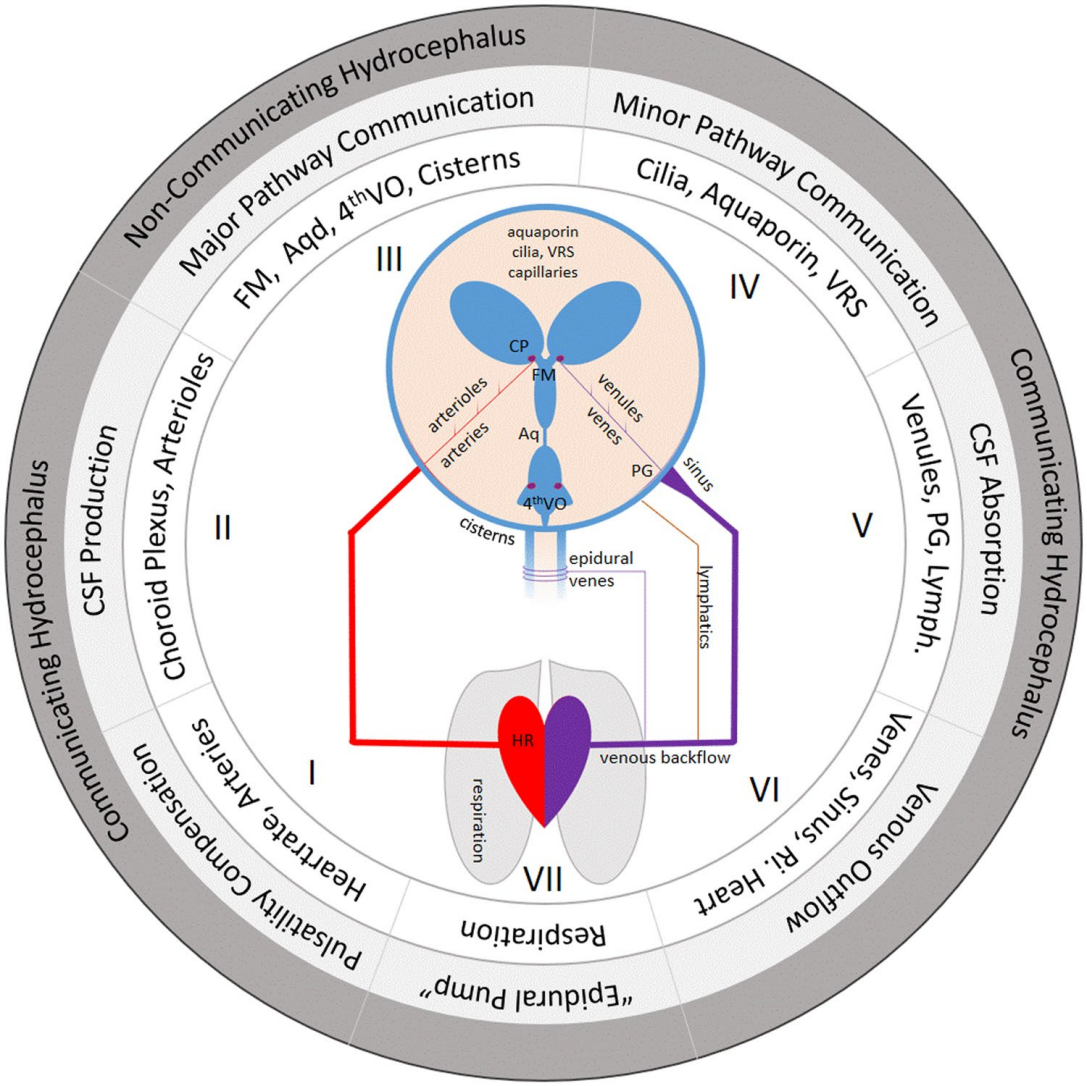
Seven factors which may be relevant in hydrocephalus pathogenesis: I: decompensation of pulsatility, II: CSF overproduction, III: major pathway obstruction, IV: minor pathway disturbances, V: CSF absorption disturbances, VI: venous congestion, VII: respiratory imbalance. Single factors may be predominantly responsible or different ones may contribute to hydrocephalus genesis. (Abbreviations: CSF, cerebrospinal fluid; FM, foramen of Monro; Aqd, aqueduct; 4thVO, 4th ventricular outlets; VRS, Virchow Robin spaces; PG, Pacchioni granulations; Ri, right; HR, heart rate; CP, choroid plexus)

**Fig. 2:**
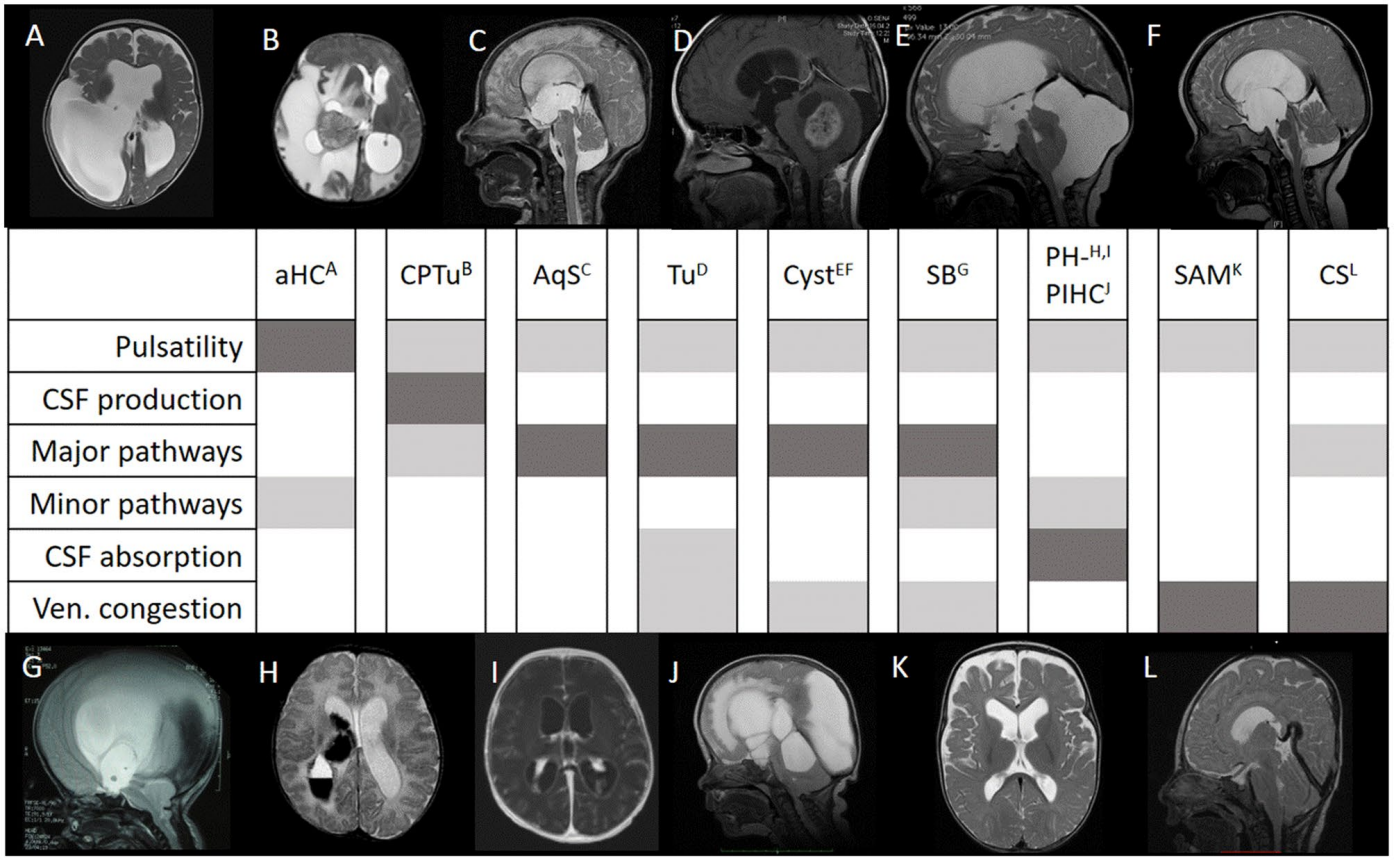
Six of seven known factors to contribute to different entities of hydrocephalus for its development. The major factors mainly causing the hydrocephalic condition are depicted in dark gray while possible contributing factors are given in light grey. It would not mean that other factors could also contribute to the hydrocephalus during the time course of the disease, but it should give a general overview of the concept of interaction among the factors in the individual case. Hydrocephalus entities are **A** arrested hydrocephalus (aHC); **B** choroid plexus tumor (CPTu); **C** aqueductal stenosis (AqS); **F** posterior fossa tumor (Tu); **E** retrocerebellar cyst (Cyst); **F** suprasellar arachnoid cyst (Cyst); **G** myelomeningocele/spina bifida–related hydrocephalus (SB); **H** posthemorrhagic hydrocephalus (PHHC) **I** ventriculitis/postinfectious hydrocephalus (PIHC) **J** multi-loculated hydrocephalus (MLHC); **K** subarachnomegaly (SAM), macrocephaly; **L** craniosynostosis (CS)-associated hydrocephalus

Different theories have been defined how hydrocephalus may develop. In recent years, older theories are challenged, and additional explanations are made in order to understand the unified theory for the pathophysiology of developing hydrocephalus. However, it might not be relevant to identify the one and only theory but rather to understand the complex nature of CSF circulation disturbances and respect the different natures of disturbances which will lead to the individual hydrocephalic condition. On the basis of the hydraulic circuit by Rekate, the interaction between blood flow and CSF circulation are described [65]. The model however could be expanded by adding the factors of pulsatility alterations, intraparenchymal CSF circulation (minor CSF pathways), the lymphatic resorption pathways, and the interaction with respiratory rhythm (Fig. 1). Using such a model, seven pathophysiological contributing factors for CSF circulation disturbances may be defined. These factors may exclusively be relevant to explain certain hydrocephalus entities but will more often be responsible in combination with each other to contribute pathological CSF flow disturbances in the individual case. It is our task to better understand different diseases causing hydrocephalus to find the primary or combining factors being responsible for its development (Fig. 2).

Within the seven factors, the activity of the heart, which is transmitted by the arteries into the intracranial space and the parenchyma, is determining the main pulsatile movement in the CSF (I). Based on DiRocco and according to Greitz and Preuss [19, 28, 61], if the pulsatile amplitude is imbalanced and pressure peaks become harmful for the brain as well as for a physiologic free flow of CSF, the decompensated equilibrium of CSF pulsatility is leading to conditions mostly seen in normal pressure hydrocephalus or decompensation of arrested hydrocephalus but may also be contributing to any kind of hydrocephalic state. CSF production (II) has been proven to originate from the choroid plexus but also from the proximal high-pressure capillary system in the ependyma as well as in the parenchyma. The fact that hydrocephalus occurs more likely in choroid plexus papilloma than in any other type of brain tumor during childhood keeps the emphasis on the role of choroid plexus for CSF production [60]. On the other hand, choroid plexus coagulation will not necessarily lead to complete inhibition of CSF secretion, underlining the fact that other resources of CSF production exist [44–46]. The relevance of major CSF pathway (III) is obvious since Dandy distinguished between communicating and non-communicating hydrocephalus and is still somehow valid until today giving us the indication of endoscopic fenestration of membranes and reestablishing CSF communication among the CSF spaces. Dandy already proved the theory by giving dye into the lateral ventricle, and it quickly appeared in the spinal CSF as seen in lumbar puncture [18]. More recent radionuclide tracers could prove the fast communication of substances along the major CSF pathways into the spinal canal within minutes [39]. We see a lot of major CSF obstruction by tumors, cysts, aqueductal obstruction, or others leading to non-communicating hydrocephalus, which does not mean that all obstruction of major CSF pathways will lead to hydrocephalus since possible compensating mechanisms of minor CSF pathways and intraparenchymal CSF absorption may keep a compensated state in some of the cases [13, 57]. Why one is behaving differntly to the others will need further intensified research. The fact that Oreskovic and colleagues could not find CSF outflow from the aqueduct in their experimental setting [40] is in contrast to rapid distribution of any dye from the ventricle into the spinal CSF spaces which might be explained due to the fact that the setup was performed in pure horizontal position, and the externalization of the aqueductal tubing did exclude any pressure relations to external CSF spaces which might be an indispensable factor for CSF communication during respiration as well as during posture changes. Minor CSF pathway obstruction (IV) is maybe the most complex cause of CSF circulation disturbances since many different microscopic structures are involved in trans- and intraparenchymal CSF flow [1, 32, 41, 48, 55]. This includes the ependymal and the newly discovered role of ependymal cilia for CSF micromovements. Transependymal CSF transport takes place by gradients or by aquaporin channels and the movement of interstitial water along the perivascular spaces also known as Virchow Robin spaces [41, 54, 64]. Pulsatile pressure gradient as well as osmotic gradients may be the relevant driving forces of interstitial CSF flow. The absorption of CSF (V) is enabled by different structures such as the low pressures capillaries (venules), the lymphatic pathways, and the arachnoid villi [33, 41, 53, 78]. Both minor CSF pathway as well as CSF absorption pathologies might mainly be present in congenital malformative hydrocephalus with altered microstructure of the brain as well as postinfectious and posthemorrhagic hydrocephalus, however, could also be involved in other conditions if decompensated pressure does compress the parenchyma and lead to microstructural failure. Venous outflow obstruction (VI) might be present in direct venous compression, sinus outflow occlusion, or right heart failure leading to a change in CSF venule pressure gradient with impaired CSF absorption. Classical entities are the macrocephaly of the infancy and the idiopathic intracranial hypertension [5, 9, 70]. But also, cranial constriction in complex craniosynostosis cases does develop a relevant pathophysiological factor of venous congestion leading to CSF outflow problems in some cases [15, 16]. This however might be combined with major CSF pathways obstruction e.g., of the 4th ventricular outlets caused by posterior fossa compression and secondary Chiari malformation. Finally, the respiration (VII) has shown to be a relevant factor for CSF transport from the spinal CSF space into the intracranial space which is driven by spinal epidural venous filling leading to a kind of venous pump mechanism in horizontal body position [2, 12, 21, 50]. Investigations to describe this mechanism have been performed in real-time MR imaging with forced respiration protocol in horizontal body position. It remains unclear and rather unlikely if the respiration has the same driving mechanism of CSF flow in vertical position or even in the moving body. Influences of respiration on any hydrocephalic entity need further investigation and are not well understood at the current stage. In this context, it might however be hypothesized that CSF movement is mostly pulsatile as driven by the cardiac cycle, while CSF distribution and different stages of CSF net flow within the pulsatile cycle may be driven by respiration and different body position and activity.

### Therapeutic considerations

**Fig. 3:**
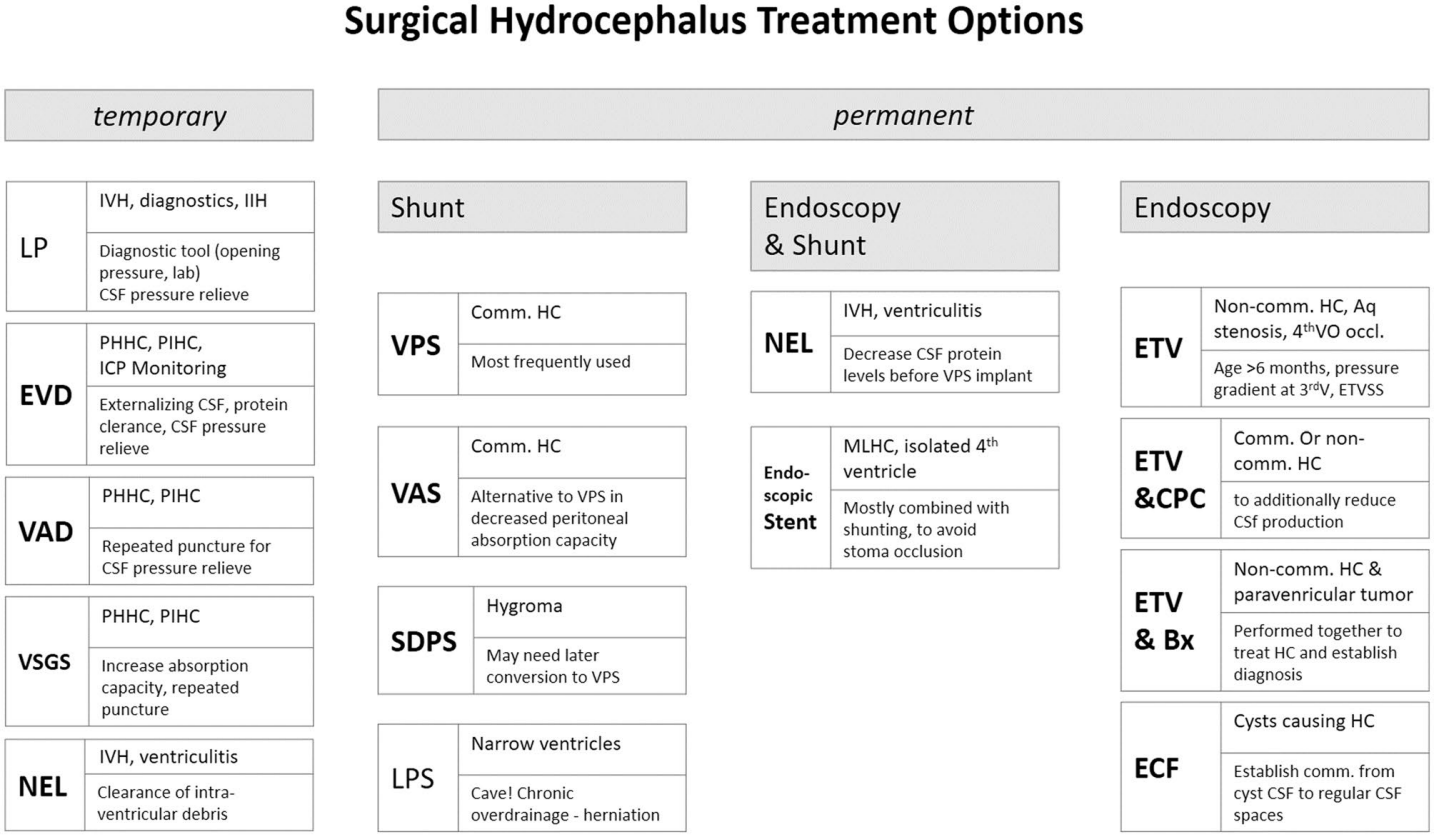
Overview of surgical treatment options for hydrocephalus in infancy and childhood categorized in temporary and permanent treatment options. (Abbreviations: LP, lumbar puncture; EVD, external ventricular drainage; VAD, ventricular access device; VSGS, ventricular subgaleal shunt; IVH, intraventricular hemorrhage; IIH, idiopathic intracranial hypertension; CSF, cerebrospinal fluid; PHHC, posthemorrhagic hydrocephalus; PIHC, postinfectious hydrocephalus; ICP, intracranial hypertension; VPS, ventriculoperitoneal shunt; VAS, ventriculoatrial shunt; SDPS, subduroperitoneal shunt; LPS, lumboperitoneal shunt; comm., communicating; non-comm., non-communicating; HC, hydrocephalus; NEL, neuroendoscopic lavage; MLHC, multi-loculated hydrocephalus; ETV, endoscopic thirdventriculo(cisterno)stomy; CPC, choroid plexus coagulation; Bx, biopsy; ECF, endoscopic cyst fenestration; Aq., aqueduct; 4thVO, 4th ventricular outlets; ETVSS, ETV success score; Tu, tumor)

In contrast to a rather complex classification of pathophysiology, the therapeutic options we apply as pediatric neurosurgeon are rather limited but are still a challenge to apply in the optimal way. Temporary and permanent treatment options of hydrocephalus are distinguished (Fig. 3).

Among temporary treatment options, there are CSF punctures, external ventricular drainage, ventricular access device, or ventricular subgaleal shunt. For CSF puncture, transfontanelle puncture is reserved for emergency situations and becomes obsolete due to higher rate of infectious complications [77]. Lumbar puncture is infrequently used for CSF relief in neonates or regularly applied for diagnostic purposes in IIH. It must be respected that only external CSF spaces may be drained in non-communicating hydrocephalus, which might lead to increased pressure gradient between internal and external CSF spaces and may cause clinical deterioration due to trans foramen magnum herniation [9, 81]. External ventricular drainage might be used for externalizing protein or bacteria polluted CSF out of the ventricular system. It is also important for draining CSF and measuring ICP in parallel especially in cases with impaired consciousness. EVD in neonates, however, is associated with a higher grade of reoperations in neonates with IVH. Ventricular access device (VAD) and ventricular subgaleal shunt (VSGS) are mainly used in IVH associated with hydrocephalus. While VAD is used for safe and sterile repeated CSF punctures, VSGS is meant to reduce the amount of punctures needed by increasing the CSF absorption capacity in the subgaleal pocket [7].

Permanent hydrocephalus treatment consists of shunting, endoscopic procedures, or both may be combined. Shunt procedures still account for the vast majority of permanent hydrocephalus treatments, and ventricular peritoneal shunting is used most frequently. Alternatives such as ventricular arterial shunts or others are used when the resorption capacity of the peritoneum is impaired due to e.g., peritonitis or necrotizing enterocolitis in the neonates. Subdural-peritoneal shunts are used in hygroma-associated CSF circulation disturbances and may need removal or conversion into VP shunt if hydrocephalus persists. Cystoperitoneal shunts should be avoided since almost all cysts do not represent a hydrocephalic condition but rather a problem of CSF isolated compartments, which should be broad in communication with regular CSF spaces by endoscopy or microsurgery [6, 38]. Lumbar peritoneal shunts are described for circumstances of narrow ventricles but represent a rather problematic solution especially in children since frequently seen chronic over-drainage in this age group may lead to secondary Chiari Malformation with possible herniation in the long run [14]. In general, the shunt itself is a non-physiological CSF drainage into another body compartment, which is one of the most successful treatments in Neurosurgery on the one hand but still is associated with significant complication and reoperation rates. Complications such as malpositioning, obstruction, infection, and over- and under-drainage need full awareness for the treating pediatric neurosurgeon in order to find solutions to keep the complication rate as low as possible. Since pathophysiology of different types of hydrocephalus is so diverse as well as activity, age, height, growth, and anatomical circumstances are individually so heterogeneous, the adaptation of resistance in the valve system per case and over time seems to be still underestimated [24, 30]. Moreover, underdrainage mostly exists when a shunt is somehow obstructed; over-drainage is a common problem infant and childhood hydrocephalus will develop over time, e.g., already when the patient gets in the upright position and the calvarial cavity get in a higher position compared to the peritoneum inducing hydrostatic force in the shunt. This chronic problem needs to be addressed in the shunt system from early on to avoid long-term anatomical changes such as microcephaly or slit ventricles. Increased activity over time will further aggravate over-drainage in the developing child and needs adaptation, accordingly [30].

Among endoscopic techniques, ETV is well established in non-communicating hydrocephalus to reestablish communication between internal and external CSF spaces but may not be used under the age of 6 months due to higher failure rates. Especially in North America and Africa, ETV is combined with choroid plexus coagulation to enhance the success rate and avoid shunting [46, 67]. This is still discussed controversially, especially if alternatively, more physiological shunt systems are used to optimally reach physiological CSF equilibrium in the calvarium and thereby keeping the choroid plexus intraventricularly intact. ETV in combination with biopsy might be relevant for pineal region or other paraventricular tumors causing hydrocephalus to stabilize the acute condition and establish a diagnosis to apply the optimal tumor treatment in further course [42, 71]. Endoscopic lavage has been used for intraventricular hemorrhage as well as ventriculitis and hydrocephalus. It has been shown to reduce shunt rate and reduce shunt complications over time if applicable by reducing CSF protein levels [10, 17, 23, 74]. Further studies are on the way in order to better define its role in the hydrocephalus treatment protocols [79]. Endoscopic stenting together with shunting is used especially if communicating hydrocephalus is present in addition with isolated, non-communicating compartments such as multi-loculated hydrocephalus after infection in neonates or an isolated 4th ventricle. Here, it is of utmost importance to combine navigation with endoscopy in order to optimally plan and place the stents and ventricular catheters [73, 75]. In summary, endoscopy has become more and more important in pediatric hydrocephalus treatment, and basically, everybody who takes surgically care of children with hydrocephalus should be or become an expert neuroendoscopist.

## Conclusion

Pediatric hydrocephalus is a complex disease, and deep knowledge is necessary to better understand each individual problem we are facing in our routine clinical work. The described seven categories of pathophysiology is a practical approach which might be applied for each patient in order to better understand the individual condition of hydrocephalus. Those may be existent alone or more often in combination in different types of hydrocephalus. Those will also help to better apply the optimal surgical treatment. Further research, however, is still of utmost importance to develop better protocols to reduce complication rates over time and enhance the neurocognitive outcome of our patients.
